# Transnational Solidarity Within the EU: Public Support for Risk-Sharing and Redistribution

**DOI:** 10.1007/s11205-022-02937-2

**Published:** 2022-05-27

**Authors:** Ann-Kathrin Reinl

**Affiliations:** Geschwister Scholl Institute of Political Science (GSI), Chair of Comparative Political Science, Ludwig-Maximilians-Universität Munich, Oettingenstr. 67, 80538 Munich, Germany

**Keywords:** Solidarity, Risk-sharing, Redistribution, European Union, Multi-group confirmatory factor analysis, MPLUS

## Abstract

This paper aims to discover underlying, as yet theoretically and empirically unexplored, distinctions in citizens’ views of transnational solidarity within the European Union (EU). Building on literature regarding national welfare states, the paper presents an original concept of transnational solidarity consisting of two latent, not directly measurable, dimensions: first, citizens’ preferences for risk-sharing among EU states and, second, their preferences for intra-EU redistribution. The diverse types of transnational solidarity examined in previous research should be assignable to either one or the other dimension. Moreover, previous research is based on the idea that the concept of transnational solidarity is comparable across EU countries; however, this premise has not been empirically examined so far. To test both these assumptions, I analyze data collected in Austria, Germany, and Greece in 2019 or 2020. The study runs multi-group confirmatory factor analysis to test whether the presented concept of transnational solidarity (a) applies and (b) is comparable between these nations. The empirical analysis supports both these assumptions. The populations of the three countries share the same understanding of transnational solidarity even though the willingness to express cross-country risk-sharing and redistribution varies significantly between the states. The study contributes to current research in the fields of European integration, political sociology, and survey methodology.

## Introduction

Every community needs a certain degree of solidarity to exist (Ferrera, 2017: 15). The European Union (EU) is no exception and, following more than a decade of crises, solidarity between EU member states has often been called for, especially compared to non-crisis periods and to any other period in the organization’s history. In the aftermath of the so-called European sovereign debt crisis,^1^ EU member states and citizens have been divided over whether to provide financial assistance to fellow states suffering from monetary and economic difficulties. In addition, the EU faced a second continent-wide challenge, which partly overlapped the financial turbulences in time. In 2015 and 2016, more than 2.5 million refugees entered the EU (Eurostat, 2020a). This so-called “migration crisis” represented a further test for the weakened union and for solidarity between its member states. During both these crises, a gap between demand and supply of solidarity within the EU became apparent, where the demand side outweighed the supply side (Genschel & Hemerijck, 2018: 2).

Previous scholarly work has shown that, depending on the underlying concept of solidarity used in each study, divergent results might appear regarding the level of public support for intra-EU solidarity. Whereas Lahusen and Grasso (2018)^2^ only find limited public backing for EU-wide solidarity actions, Gerhards and colleagues^3^ (2019) show that the majority of EU citizens favor transnational solidarity. Defining and operationalizing solidarity is therefore key to understanding EU-wide public support for this phenomenon.

Preceding studies that aimed to disentangle the concept of solidarity in the EU context have empirically differentiated between public support for solidarity in various policy areas and crisis scenarios (Baute et al., 2018; Baute et al., 2019a, b; Gerhards et al., 2019; Katsanidou et al., 2021; Lahusen & Grasso, 2018). However, despite these studies, research still lacks an overarching concept of individual-level EU solidarity, which is referred to as transnational solidarity in this work. This hinders us from understanding how earlier findings in the field are interrelated. *How do the operationalizations and measurements of transnational solidarity made in previous studies link to each other, and is it possible to discern more coarse-grained and hitherto unidentified patterns underlying them?*

A comprehensive concept covering previously studied dimensions of transnational solidarity is hence a necessary next research step. In order to move away from situation-based and context-dependent definitions, a concept capable of being applied to a multitude of past and future crises and policies is needed. This is relevant not only for investigating the current state of transnational solidarity in the EU population, but also for attempting to draw more far-reaching conclusions about future trends based on such a comprehensive approach.

In line with this reasoning, this study follows a theoretical approach usually applied in national welfare state research and transfers it to the EU level. Citizens are presumed to have similar reflections at both political levels. To be more precise, the paper tests for the existence of two underlying dimensions of transnational solidarity that cut across previous classifications: risk-sharing provided for fellow EU member states in crisis, as well as redistributive policies striving for more strongly embedded solidarity in the overall European integration process. The diverse aspects of transnational solidarity examined so far should be assignable to either one or the other dimension and should consequently be reflected in this two-dimensional concept. Moreover, I test whether and to what extent the two derived dimensions of solidarity speak to each other.

In addition, measurement equivalence between EU countries is often assumed but seldom tested (Kankaraš & Moors, 2009: 557). This marks a clear shortcoming in the current field of transnational solidarity research. Cross-border measurement invariance is a prerequisite for theoretical concepts to be empirically compared across different groups. Otherwise, there is a risk that latent and not directly measurable concepts will have a different meaning for one group or country than for another (Cieciuch et al., 2016; Davidov et al., 2014). Following this line of argument and since the EU is a union of countries that are heterogeneous in many respects, it is important to ensure that the concept studied is genuinely applicable across borders.

For this purpose and to contribute to both identified gaps in the literature, I analyze survey data collected in Germany and Greece between June 2019 and August 2019 as well as in Austria in January 2020, following the peaks of the European sovereign debt crisis and the so-called migration crisis. Multi-group confirmatory factor analysis is performed in MPLUS in order to test whether the presented concept of transnational solidarity (a) applies and (b) is comparable between EU member states. The analysis reveals that the concept indeed exists and is comparable across the nations being investigated. Citizens in the three countries share the same understanding of transnational solidarity even though the general willingness to express solidarity with fellow EU member countries and EU citizens varies significantly among the groups. In other words, despite similar understandings of what transnational solidarity means, people in the three countries studied express varying levels of preferences for actively demonstrating such solidarity. These are important findings both for academia as well as for policymaking. Risk-sharing and redistribution policies might gain (even more) relevance in the context of future EU-wide challenges. The empirically verified two-dimensional concept of transnational solidarity presented in this work can thus help in predicting public backing for upcoming policy proposals and the shaping of a more solidarity-oriented union. The study theoretically and empirically expands the state of knowledge in the fields of European integration, political sociology, and survey methodology.

## Transnational Solidarity in the European Union

Solidarity is a fuzzy concept (Grimmel, 2021; Lahusen, 2020; Wallaschek, 2020) and therefore a clear demarcation is important to distinguish it from related ideas. Solidarity is not charity (Sangiovanni, 2018) and sometimes not even altruistically motivated (Stjernø, 2011). Instead, “[s]olidarity is costly. […] Solidarity involves sharing in a real, material sense” (Genschel & Hemerijck, 2018: 2). Responsibility for one another (Lahusen, 2016: 8) and mutual dependence between individuals lie at the heart of every solidarity-oriented action (Lahusen, 2020; Sangiovanni, 2018: 17; Stjernø, 2011: 168–169). Thus, solidarity is not a one-way street. Instead, “[s]olidarity is symmetrical, reciprocal, and omnilateral not asymmetrical, non-reciprocal, and unilateral” (Sangiovanni, 2018: 24).

In the context of the EU, Lahusen (2020: 11) claims that “European solidarity can be defined as an attitude and behaviour in support of other Europeans, regardless of their national origin”. Gerhards et al., (2018: 6) not only focus on citizens as recipients of solidarity-based actions, but also consider the country level in their definition: “By European solidarity, we understand a form of solidarity that goes beyond one’s own nation state, and where the recipients of solidarity are other EU countries, or citizens of other EU countries”. This paper uses the definition developed by Gerhards et al. (2018) and focuses on investigating citizens’ preferences for EU-wide solidarity measures. To be more precise, I do not analyze whether citizens genuinely act in solidarity but whether their preferences show “signs of solidarity” (Gerhards et al., 2019: 23) or “active support” for it (Lahusen, 2020). The term European solidarity is, however, generic and does not allow conclusions to be drawn about the level of observation (that is, whether individuals or larger entities are being examined). Consequently, and in order to make a statement about the donor side of solidarity, I will refer to this type of solidarity as transnational solidarity in the context of the EU.

Solidarity constitutes one of the core values of European integration (Sangiovanni, 2013: 213) and is explicitly mentioned in various EU treaties. Put into practice, solidarity has been implemented to various degrees within the EU depending on the issue area. It is strongest in socio-economic terms and still rather weak with regard to, for instance, environmental policies (Domurath, 2013). During the European sovereign debt crisis, new mechanisms were implemented to financially assist debtor states. The treaty establishing the European Stability Mechanism (ESM) was set up as a persistent source of financial assistance in September 2012 and refers to responsibility and solidarity within the Eurozone (ESM, 2020). During the years of high migration numbers, the opening of the German and Swedish borders to refugees was interpreted as a sign of solidarity, as it briefly undermined the Dublin Regulation and relieved the burden on countries at the EU’s external borders (Steinvorth, 2017: 12–13). This shows that solidarity in the EU context can have many facets and is often evoked in response to immediate crises. Beyond this, and independent of any acute crisis scenarios, the funds set up by the EU to promote economic convergence across regions represent another practical implementation of EU-wide solidarity measures (European Commission, 2020).

In empirical terms, transnational solidarity is a latent concept that cannot be measured directly by means of a single indicator but consists of various dimensions that become apparent depending on the respective situation and policy field. Literature studying solidarity in the context of the EU has already tried to disentangle the latent concept of transnational solidarity with its various dimensions (for instance Gerhards et al., 2019; Lahusen & Grasso, 2018). Nonetheless, and despite laudable efforts, most of these studies did not relate the single dimensions of transnational solidarity to each other but examined them separately. Until today, only a handful of studies have considered the interplay between individual dimensions of transnational solidarity. Baute and colleagues (Baute et al., 2018; Baute et al., 2019a, b) have published several studies in which they disentangle public preferences for the latent concept of “Social Europe” that is linked to the idea of transnational solidarity referred to in this paper. In addition to dimensions of transnational solidarity, the concept used in their work addresses aspects of European social citizenship and the level of decision making. Their empirical findings based on confirmatory factor analysis indicate that, Social Europe consists of various aspects that cannot be limited to a single factor but are instead interrelated.

Following this, although a limited number of preceding studies has analyzed and connected the various dimensions of transnational solidarity, research on transnational solidarity carried out so far has consistently analyzed support for solidarity in the context of particular crisis scenarios or policy areas without agreeing upon whether there are underlying dimensions that give rise to similar results. Whereas in recent European crises it has been important to focus on each individual crisis separately and to explore specific forms of solidarity, it is now time to move a step further and to reveal existing, yet unexplored, dimensions that underlie the individually measured indicators and to relate them to each other. There is a need for a more all-encompassing conception of transnational solidarity that brings together the existing pieces of the puzzle—not only from an academic perspective, but also with regard to current policymaking. The aim is to develop a comprehensive concept that incorporates previously applied operationalizations into its framework, and at the same time remains coarse-grained enough not to become mired in a multitude of situation-specific dimensions. Such an approach would allow for broader predictions about current and future societal dynamics to be made. The guiding research question therefore asks: *How do the operationalizations and measurements of transnational solidarity made in previous studies link to each other, and is it possible to discern more coarse-grained and hitherto unidentified patterns underlying them?*

My attempt to address this research question draws on a distinction usually made in national welfare state research. Referring to this strand of literature is justifiable considering that solidarity in the nation-state context has already been extensively researched. The EU represents an overarching layer in the EU’s multi-level political system, and has become increasingly involved in its member states’ affairs over the years. Consequently, it is logical to assume that EU citizens adopt similar patterns of reasoning when it comes to understanding and evaluating social policies at differing political levels (Gerhards et al., 2019). This assumption has already been backed by a study from Ignácz (2021), in which she compares levels of endorsement for redistributive policies at the national and EU level and identifies a strong overlap between the two levels. Conversely, this would not be expected to apply to levels of regional or international policies, as these generally do not guide state-led redistribution and risk-sharing programs.

Vandenbroucke (2020: 8) uses the term “‘welfare state solidarity’ as an umbrella concept for redistribution and insurance”. Both these types of welfare state solidarity are geared towards making a society fairer, but they do so in different ways (Esarey et al., 2012: 686; Rothstein, 1998). Insurance, or risk-sharing, follows an insurance logic and provides protection against crisis situations whenever unforeseeable events occur (Esarey et al., 2012; Pettersen, 1998; Vandenbroucke, 2020). In contrast, redistribution aims to systemically fight inequality, regardless of whether a crisis is happening (Esarey et al., 2012; Roller, 1998; Vandenbroucke, 2020). The following sections of the paper apply this distinction between risk-sharing and redistribution to the EU level to assess whether transnational solidarity might be represented by a similar umbrella concept.

## The Two-Dimensional Concept of Transnational Solidarity

The EU’s various crises over the last decade have served as tests for how to act practically in solidarity (Grimmel & Giang, 2017: 2). In times of economic crisis, for example, welfare state policies can help to overcome economic decline by putting into place so-called “automatic stabilizers”, designed to absorb shocks as effectively as possible (Vandenbroucke, 2017: 157, 2018: 8). Crisis aid is mostly a short-term measure designed to deal with a crisis at its acute stage and, if necessary, to carry out reconstruction. It could also, under certain circumstances, evolve into a longer-lasting aid program. The policy instrument of risk-pooling, for instance, is not a short-term instrument as it manifests itself in an institutionalized form of solidarity. Whereas EU redistribution policies aiming to tackle economic inequality might reduce in importance over time because economic standards are increasingly converging,^4^ so-called risk-sharing measures never lose their topicality since crises can disrupt a community at any time (Vandenbroucke, 2020: 36–37).

In the context of the EU, these kinds of risk-sharing policies mostly occur in the form of financial assistance provided for the entity in crisis, which is particularly relevant in the context of the European monetary union (Schelkle, 2017). Previous studies have analyzed public support for financial assistance provided in scenarios of financial and economic difficulties (Díez Medrano et al., 2019; Ferrera & Pellegata, 2017; Genschel & Hemerijck, 2018; Gerhards et al., 2019; Lahusen & Grasso, 2018), high numbers of incoming migrants (Genschel & Hemerijck, 2018; Gerhards et al., 2019; Lahusen & Grasso, 2018; Reinl, 2020) or the occurrence of natural disasters (Díez Medrano et al., 2019; Genschel & Hemerijck, 2018). However, no study so far has combined these various indicators of crisis-related transnational solidarity, so there is yet no evidence to suggest whether people would in fact be willing to bear a joint risk for an EU partner state and its population, regardless of the nature of the crisis.

EU citizens are considered to weigh up the costs and benefits of solidarity-based emergency aid in any possible crisis scenario. Rational cost–benefit calculations characterize all types of crisis aid and thus, risk-sharing should form one overarching dimension of the latent concept of transnational solidarity. With any kind of crisis aid, the donor side does not expect immediate compensation from the recipient party. Even if financial aid is conditional, it remains open when and to what extent a reciprocal aid scenario would occur. Consequently, the donor side invests financial resources in any crisis scenario affecting another EU member state with little evidence as to whether this aid is financially worthwhile for the donor’s own country. Thus, the risk of crisis is borne on several shoulders, at least in the short term.

Because public support for financial crisis assistance is expected to follow the same logic regardless of the origins of a crisis, risk-sharing should form one overarching dimension of the latent concept of transnational solidarity. This by no means assumes that the willingness to show solidarity is the same for every crisis, but that every type of crisis aid addresses a common theoretical dimension of transnational solidarity. This assumption is supported by a more recent publication showing that despite diverse levels of support for EU risk-sharing depending on the crisis scenario (economic, migration-related, Covid-19), the underlying drivers for it remain almost identical (Katsanidou et al., 2021).

### H1

Indicators measuring support for EU risk-sharing are part of one underlying dimension of transnational solidarity.

The second presumed dimension of transnational solidarity refers to more permanent redistribution. It relates to the idea of integrating solidarity into the EU integration process and viewing solidarity as a higher purpose of the community. Within the European Union, this encompasses all kinds of policies that aim at reducing inequalities between EU member states and their populations in the long run and which do not pursue the temporary goal of supporting another state when a particular crisis occurs (Vandenbroucke, 2020). What would such redistributive practices look like? On the one hand, one could think of long-term oriented financial transfers to economically weaker EU regions (Ignácz, 2021). Examples of this are the European Structural and Investment Funds designed to enhance economic development and convergence between EU member states (European Commission, 2020). On the other hand, one could propose setting up a EU-wide welfare state. Even though the economic systems of EU nation states have become increasingly intertwined, this development has not occurred in the realm of welfare state policies (Ferrera and Pellegata, 2017; Pantazatou, 2015: 54–55). Consequently, as transnational solidarity in terms of permanent redistribution is intended to make the community fairer in the long run, its various facets are expected to address a second overarching dimension.

### H2

Indicators measuring support for EU redistribution are part of one underlying dimension of transnational solidarity.

The aim of this study is to get as close as possible to an all-encompassing concept of transnational solidarity within the EU. Acknowledging the multidimensionality of a concept is important for the scholarly debate because otherwise researchers risk overlooking distinct mechanisms and characteristics underlying the particular individual dimensions (Lomazzi, 2021). The diverse types of transnational solidarity studied in previous research should be assignable to either the risk-sharing dimension or the intention of making the EU more solidarity-oriented over the longer term by way of redistributive policies. This distinction between these two types of solidarity-oriented policies is usually made in national welfare state literature (see for instance Vandenbroucke, 2020) and citizens are assumed to have similar mindsets with regard to related policies at the EU level (Gerhards et al., 2019; Ignácz, 2021). The two dimensions should not be mixed together to reflect transnational solidarity as one dimension. Instead, since both dimensions embody different facets of transnational solidarity, I argue that transnational solidarity is a two-dimensional concept with both dimensions being separate but interrelated (covarying). This theoretically derived two-dimensional concept of transnational solidarity should also be empirically identifiable within EU member states:

### H3a

Transnational solidarity consists of two dimensions with one dimension capturing EU risk-sharing and another one capturing support for EU redistribution.

Despite familiar differences between EU member states in terms of public support for financial assistance in crisis scenarios (Domurath, 2013; Lahusen & Grasso, 2018) as well as regarding more deeply rooted attitudes towards redistribution (Gerhards et al., 2016), the proposed concept is presumed to equally represent transnational solidarity across EU member states. Evaluating cross-country comparability of a theorized and empirically measured construct is of utmost importance, but has so far been neglected in the research field. Numerous studies have compared transnational solidarity across national borders, without testing for how it is understood by people across borders.^5^ This can lead to false inferences being made, as it cannot be ruled out that phenomena or individual components of it are defined and interpreted differently depending on national contexts (Davidov et al., 2014).^6^ A welcome exception to this is the work of Lomazzi (2021; for a similar approach based on older data see also Kankaraš & Moors, 2009), which tests for the comparability of solidarity across European (including non-EU) countries. She concludes that the understanding of solidarity among people across countries is broadly similar with regard to most but not all of its dimensions.^7^ However, solidarity is a rather broad concept in Lomazzi’s and does not explicitly address the EU level,^8^ which leaves a research gap. In view of the two-dimensional concept of transnational solidarity presented here, I expect to find comparable understandings across national borders. I assume this, since preferences for transnational solidarity, regardless of the levels of support in the respective states, should still be assignable to one of the two dimensions presented.

### H3b

The understanding of transnational solidarity is comparable between citizens of different EU member states.

## Data and Operationalization

The analysis makes use of a novel data collection in three EU member states depicting extreme interests during the European sovereign debt crisis and the years of high migration numbers: Austria, Germany, and Greece. Evaluating extreme cases allows the widest possible range of countries to be analyzed without necessarily requiring information on every member of a sample. If similar results are found in the analysis of very different cases, the findings can most likely also be applied to other cases in the sample. In order to finally confirm this, it is necessary to verify the results in future analyses using data from other EU countries. At present, however, there is no publicly accessible data source that would allow the concept of transnational solidarity drawn here to be adequately measured over a larger number of EU member states. Addressing this lack of data would constitute a fruitful starting point for future comparative surveys.

In terms of funding, Germany was the largest donor country during the sovereign debt crisis, while Greece received the largest bailouts from other EU member states. Austria also represents a donor state during the sovereign debt crisis and the country weathered the crisis well in macroeconomic terms.^9^ In contrast, over 2015 and 2016, all three nations hosted a large share of refugees and their governments hoped for more assistance to be forthcoming from other member states.^10^ In addition to crises’ intensities, while (West) Germany was a founding member of the European Community (1958), Greece (1981) and Austria (1995) followed in later enlargement rounds. This disparity in length of membership allows to draw conclusions as to whether the concept of transnational solidarity presented in this paper is also comparable across states with varying lengths of EU membership.

In Austria, data was collected via the online panel study of the Austrian National Election Study (AUNTES) in January 2020 (wave “13”) (Aichholzer et al., 2020). In Germany, the survey was conducted via the GESIS Panel (GESIS, 2020) from June to August 2019 (wave “gc”) and carried out using both online (web-based) and offline (mail) interviews. In Greece, a telephone survey was done in June 2019 (Katsanidou & Reinl, 2020) and the poll was funded by the Leibniz Research Alliance “Crises in a Globalized World”. The set of variables featured in the surveys was specifically designed to measure transnational solidarity. The variables presenting diverse aspects of transnational solidarity have all been used in previous studies and were brought together for the first time as part of these surveys. This allows capturing the variables’ interplay and to test for the existence of overarching dimensions. Due to cost and time constraints, it was not possible to incorporate in the polls every pertinent question, but the aim was to include at least three questions for each presumed dimension, focusing on different crises and redistribution aspects. In addition to the variables used in this work, further questions were asked in the polls,^11^ but these are not expected to influence the respondents’ answering behavior in any way.

After data cleaning, the analysis sample contains 2,897 participants from Austria, 3,661 from Germany and 1,198 interviewees from Greece. Risk-sharing is assessed using three survey questions regarding financial assistance in times of (1) a national bankruptcy, (2) a high influx of migrants into a country^12^ and (3) a natural disaster. The items do not refer to existing instruments like the ESM, but ask more generally about people’s willingness to support the giving of financial aid in times of crisis. This more generic formulation has the advantage that past experiences, in some cases negative, with actual political measures can thus be minimized in their effect.

To measure citizens’ preferences for redistribution, the study uses three variables which, following the logic of a Guttman scale, become increasingly specific in their wording. The first question asks about the general agreement on whether solidarity between states should be an important goal of the EU. The second question is about support for financial redistribution between EU countries, even if this means that richer countries have to contribute more. And the third variable asks respondents for their attitude towards the introduction of a European welfare system, even if this might lead to increased taxes.

More detailed information on the question wordings can be found in the appendix (Table 1). All variables are measured via four-point Likert scales. The response categories “don’t know/ don’t answer” were classed as missing values. For a better interpretation of the results, all variables were recoded so that the highest agreement is assigned the highest value. Table 2 in the appendix provides an overview on the respective sample characteristics,^13^ and Table 3 shows the correlations between the various indicators.

To empirically test the formulated research hypotheses, multi-group confirmatory factor analysis (MGCFA) via MPLUS (version 8.4) was carried out to evaluate whether the empirical data speaks to the theoretically derived dimensions of transnational solidarity and to ascertain whether the understanding of the concept of transnational solidarity is comparable between citizens of different EU countries (see for instance Brown, 2006; Davidov et al., 2014). For the handling of missing data, instead of opting for list-wise deletion, the analysis uses full information maximum likelihood (FIML), which allows including respondents in the analysis who did not provide information on every item.^14^

## Results

### Descriptive Results

Before turning to the paper’s MGCFA, I first descriptively present the dimensions of transnational solidarity in each country. Figure 2 in the appendix visualizes public support for risk-sharing in the three countries under investigation (see also Table 2 in the appendix). For all three countries, citizens’ willingness to provide financial assistance is highest in the scenario of a natural disaster. In Germany, there is almost equal support for risk-sharing in the case of a nation going bankrupt (2.27) and a migration scenario (2.34) whereas, in Greece, public approval for fiscal solidarity is higher in the event of a national bankruptcy compared to an influx of migrants. For Austria support for financial assistance is lowest in the case of a national bankruptcy, even though support in a migration scenario is only marginally, though significantly, higher (+ 0.13). Regarding a national bankruptcy, the discrepancy between countries might be due to the countries’ different experiences of the European sovereign debt crisis. In contrast, all states accommodated a large share of refugees during the so-called “long summer of migration” (see for instance della Porta, 2018) and therefore rates of support might vary to a lesser extent in this scenario.

With regard to the redistribution dimension of transnational solidarity, Fig. 3 in the appendix graphically shows the distributions of indicators for each country (see also Table 2 in the appendix). In all three countries, support is highest for the least specific statement saying that the EU should promote solidarity within the EU. Hence, this item might also tap a different side of transnational solidarity than pure redistribution. It is also apparent that support for redistribution wanes when policies are seen as more costly (Lahusen, 2020: 2–3). In all three countries the lowest level of support is found for the introduction of a common European welfare state that might be accompanied by tax increases. In general, public backing for more redistribution within the EU is highest in Greece, followed by Germany and then Austria. This difference might be because, for instance, respondents in Austria and Germany are perhaps already more satisfied with their national welfare state arrangements and do not therefore expect the introduction of a European welfare system to improve their own situation (Gerhards et al., 2019: 154). In terms of the slight yet significant discrepancy between Germany and Austria, some studies suggest that Austrians tend to be more critical towards the EU compared to Germans (Schulmeister et al., 2019), which could also be reflected in public support for redistributive policies.

### Multi-Group Confirmatory Factor Analysis

Following these first descriptive analyses, I now look at the overall concept of transnational solidarity in the countries under investigation. It is examined whether the theoretically derived concept exists empirically in the individual countries—that is, whether both dimensions are present (H1 and H2) and whether they together form a concept of transnational solidarity (H3a). I also examine whether the concept is comparable between the countries investigated (H3b). I run MGCFA opting for measurement invariance, which is described as a necessary condition for comparing concepts across groups (Cieciuch et al., 2016; Davidov et al., 2014). Measurement invariance does not assume that values are identical across groups, but that the measurement of a concept works similarly in all groups and can thus be compared on a between-group basis (Cieciuch et al., 2016: 629).

I follow a step-by-step approach, as set out by Kleinke and colleagues (2017), to look at the extent to which the theorized model “fits” the data. This is assessed based on goodness of indices. A model is considered good if the indices exceed or undercut certain thresholds (*Comparative fit index (CFI) ≥ 0.90; Root mean square error of approximation (RMSEA) & Standardized root mean square residual (SRMR) ≤ 0.08*). In this analysis, the assumed theoretical dimensions are represented by so-called factors that allow them to be empirically measurable.

**Fig. 1:**
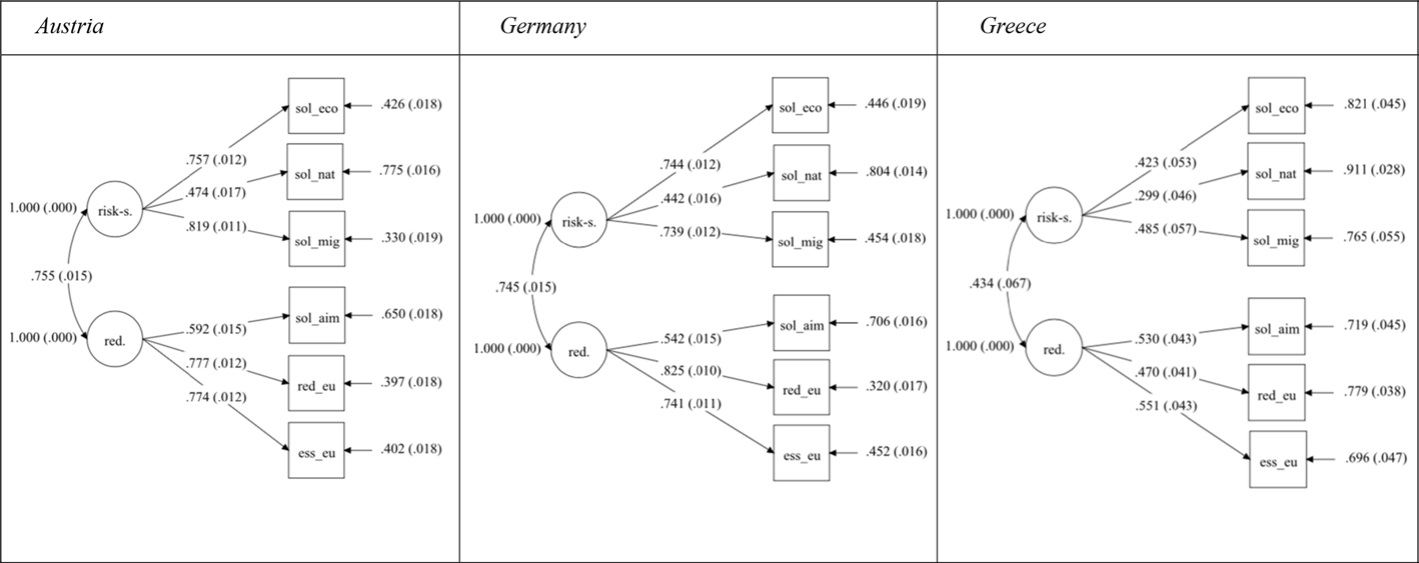
Configural model. Annotation: Estimator: ML; Parameter estimates (standardized). Model fit indices: Austria: X^2^ = 94.424, df = 8, RMSEA = 0.061, SRMR = 0.026, CFI = 0.983; Germany: X^2^ = 133.888, df = 8, RMSEA = 0.066, SRMR = 0.032, CFI = 0.977; Greece: X^2^ = 3.495, df = 8, RMSEA = 0.000, SRMR = 0.010, CFI = 1.000. All parameters are significant at the p < 0.001 level. Standard errors in parentheses. For a list of abbreviations, see Table 2 in the appendix

**Fig. 2:**
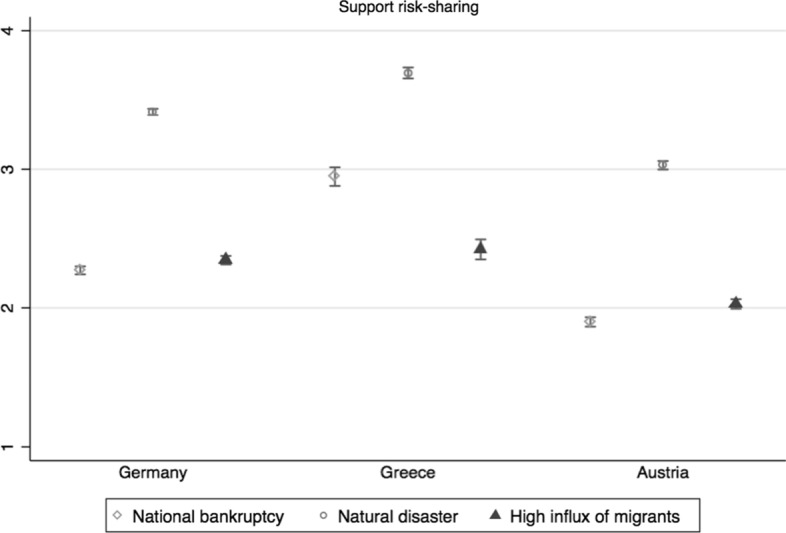
Support for risk-sharing. Annotation: Average support per country with 95% confidence intervals. All variables range from 1 (totally disagree) to 4 (totally agree). Weighted presentation: for Germany and Austria, the weightings provided by the survey programs are applied, whereas the Greek sample is weighted by age and sex

**Fig. 3:**
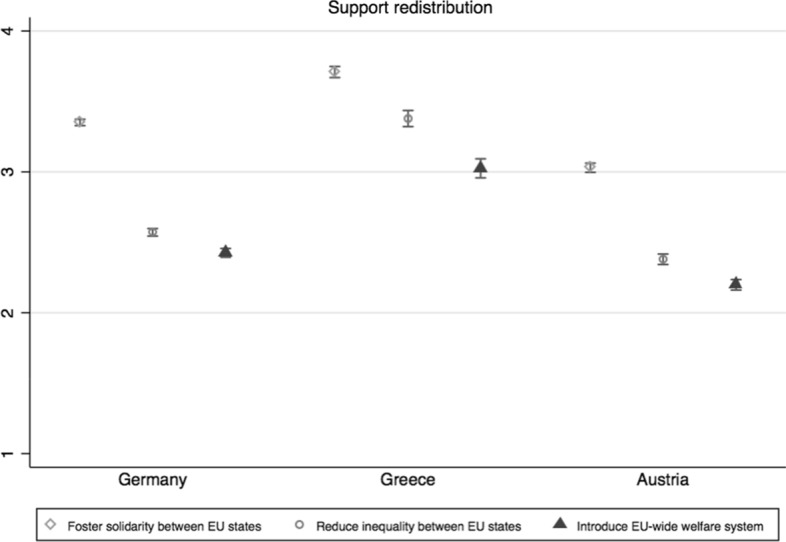
Support for redistribution. Annotation: Average support per country with 95% confidence intervals. All variables range from 1 (totally disagree) to 4 (totally agree). Weighted presentation: for Germany and Austria, the weightings provided by the survey programs are applied, whereas the Greek sample is weighted by age and sex

I will first consult the measurement models separately per country to test for the concept’s existence within the three countries. In doing so, I opt for so-called configural measurement invariance (equal number of factors and factor-loading patterns). Figure 1 graphically presents the model per country.^15^ The causal direction is from the latent factors to the items. All but one factor loading range between 0.423 and 0.825 indicating that the items are reliable indicators of the dimensions. In contrast, for the case of Greece, the factor loading for risk-sharing in times of a natural disaster is weaker, with a value of 0.299. However, since all factor loadings are at least very close to the (minimum) cutoff point of 0.3 (Brown, 2006: 130), they can still be viewed as valid indicators (see also Hooper et al., 2008). Hence, the results support the research hypotheses H1 and H2. The indicators used to capture transnational solidarity reflect the latent factors risk-sharing and redistribution. In addition, the two latent factors covary. The factors of risk-sharing and redistribution are closely linked in Austria (0.755) and Germany (0.745), whereas the relation is much weaker in the Greek case (0.434). This discrepancy between the countries could be due to the differing roles taken by the respective states, depending on the dimension. While Germany and Austria would usually take on the role of donor countries, both in terms of more permanent redistribution mechanisms and crisis aid scenarios, the situation is different for Greece. Greece has been a recipient of crisis aid over the past decade, but since the country is still in a good financial position compared to many eastern European countries, they would probably be financial contributors to any crisis-independent redistribution. Consequently, the link between the dimensions might be lower in Greece because the different dimensions would claim different roles. Whether this reasoning is accurate, however, remains unclear and cannot be evaluated conclusively within the framework of this paper.

We now know how the dimensions relate to each other and that the indicators reflect them quite well. But does the theoretically derived two-dimensional model really fit the data? For all the countries under investigation the model-fit indices (see Table 4 in the appendix) show a very good fit, indicating that the data supports the theoretically derived model. As a result, configural measurement invariance is confirmed and I identify equal-factor structures across the three EU nations studied. In line with this, the data supports research hypothesis H3a because, for respondents in all the countries investigated for this paper transnational solidarity exists of two dimensions, with one capturing risk-sharing and the other measuring support for redistributive policies across the EU.^16^ In other words, the evidence presented here suggests that both theoretically derived dimensions are present in citizens’ concept of transnational solidarity.

In the next step, and to test hypothesis H3b, I test whether the theoretical derived model of transnational solidarity also shows metric (equal factor loadings) as well as scalar (equal intercepts) measurement invariance. To evaluate whether metric or scalar measurement invariance are established, the more constrained model-fit indices are compared to the indices of the respective less restrictive models (Chen, 2007).^17^

In the event that metric invariance is established, the latent construct of transnational solidarity retrieves the same content in the three countries (Davidov et al., 2011: 152). Metric invariance allows for the comparison of regression coefficients across groups. In this case, a one-unit change in the latent construct means the same in all groups under investigation (Davidov et al., 2015: 248–249). To confirm the existence of metric measurement invariance, the model-fit indices of the metric model are compared with those of the baseline model to see whether the model significantly worsens once the factor loadings are constrained to be equal between the groups. In addition to performing chi-square difference tests, model-fit indices can be compared across models. The model-fit indices confirm the presence of metric measurement invariance (see Table 4 in the appendix). Although metric measurement invariance is not supported by the chi-square difference test,^18^ changes in the model-fit indices CFI (∆0.003), RMSEA (∆0.004) and SRMR (∆0.009) are within the permissible range (Chen, 2007). Subsequently, the latent concept of transnational solidarity presented taps the same content in Austria, Germany, and Greece.

Once metric invariance is confirmed, one can test for the existence of scalar invariance. In the case of scalar measurement invariance in addition to factor loadings, intercepts are comparable across groups, and respondents in the countries under investigation use equal scale origins (Davidov et al., 2011: 152). The model-fit indices of the metric measurement model serve as a baseline against which the more constrained scalar model is compared. I do not find full scalar invariance for my model (∆CFI = 0.050; ∆RMSEA = 0.033; ∆SRMR = 0.032). Therefore, instead of setting all factor loadings and intercepts to be equal across groups, some constraints might be relaxed (Byrne et al., 1989). MPLUS provides information about equality constraints, which might be released in order to improve the overall model fit (Cieciuch & Davidov, 2016). However, since I only count three indicators per latent factor, the scope for improving the models’ quality is limited. To reach partial measurement invariance, at least two indicators per factor must show invariance across groups (see for instance Cieciuch & Davidov, 2016; Davidov et al., 2015; Steenkamp & Baumgartner, 1998). The modification indices indicate to freely estimate two intercepts: the one for *financial solidarity in a migration scenario* and the other one for the item asking whether the *EU should promote solidarity between its member states*.

It was already shown in the work of Lomazzi (2021) that a question on solidarity towards immigrants caused problems in achieving scalar measurement invariance. This could be due to the fact that respondents in diverse countries interpret the word “migration” differently. Whereas in Greece one might think primarily of refugees, in Germany and Austria the association with the term might be stronger with regard to immigrants from eastern Europe. Regarding the question about whether the EU should promote solidarity, respondents have more room for interpretation compared with any other question. Left-wing parties, such as Greece’s Syriza party, might have framed the concept of solidarity within the EU quite differently than conservative parties, which governed Germany and Austria at the time of the survey. Consequently, respondents could have different ideas of solidarity in mind depending on the country. Whether these assumptions are accurate, however, cannot be further evaluated in the context of this analysis but opens up potential avenues for future investigations.

**Fig. 4:**
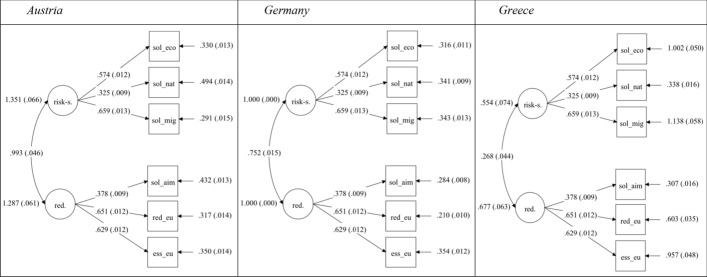
Partial scalar model. Annotation: Estimator: ML; Parameter estimates (not standardized). Overall model fit indices: X^2^ = 402.122, df = 36, RMSEA = 0.063, SRMR = 0.048, CFI = 0.966. Standard errors in parentheses. For a list of abbreviations see Table 2

As a result, I release the equality constraints for one intercept per factor. Comparing the model-fit indices (metric to partial scalar) shows that the changes in RMSEA (∆0.009) as well as in SRMR (∆0.011) are within the permissible range. For the CFI change, the value of 0.012 is only slightly higher than the cut-off point of 0.01 and so still lies within the acceptable range (Byrne & Stewart, 2006). Consequently, I find both metric and (partial) scalar measurement invariance for my theoretically derived model. This provides a sufficient basis for a reliable and valid comparison of the results across the examined EU countries. It implies that not only the factor loadings but also the intercepts are comparable across countries. Substantively, this means that citizens in the three EU countries do not only have the same understanding about the dimensions of transnational solidarity but also retrieve the same content and scales. Figure 4 in the appendix presents the partial scalar invariance model. In summary, the empirical model provides support for research hypothesis H3b: the concept of solidarity is comparable across the countries under analysis.

In addition, the achieved level of partial scalar measurement invariance now enables the mean scores of the latent factors across countries to be compared (Davidov et al., 2015). The comparison shows that the means of both factors differ significantly between the countries. With Germany as a reference group, Austrian respondents show significantly (*p* < 0.001) less agreement on both the risk-sharing (−0.759) and the redistribution (−0.347) dimension, whereas the Greek sample reports significantly (*p* < 0.001) higher mean values on the two respective dimensions (risk-sharing = 1.084; redistribution = 1.119). This implies that the populations of the three countries share the same understanding of transnational solidarity, but that the willingness to express solidarity with fellow EU member countries and EU citizens varies significantly between the groups of people interviewed in each country. It shows that Greece, as a beneficiary of solidarity-oriented policies during the past, is also the country with the most positive attitude towards it. Austria, on the other hand, is more critical of EU-wide solidarity efforts than its German neighbor, which could be due to Austrians’ generally greater skepticism towards EU integration (Schulmeister et al., 2019).

## Discussion

This paper has undertaken an in-depth analysis of the latent concept of transnational solidarity. To that end, I approached transnational solidarity through an individual level perspective using novel survey data collected in Austria, Germany, and Greece in 2019 or 2020. This study aimed to test whether transnational solidarity in the context of the European Union consists of two latent dimensions: (1) citizens’ preferences for risk-sharing and (2) public support for redistribution, and whether this concept is comparable across EU member states.

The results of my multi-group confirmatory factor analysis reveal three main things. First, the defined concept of transnational solidarity empirically holds. Second, its existence is evident in all three countries and, third, the concept of transnational solidarity presented is comparable across countries.

Why is the presence of measurement invariance an important finding for the understanding of transnational solidarity and the future of European integration? This study’s findings suggest that although there are national differences in the levels of public support for intra-EU solidarity measures, comparable understandings and interpretations of transnational solidarity among EU populations can be observed. A shared idea about the concept of transnational solidarity is desirable for several reasons. Firstly, it is important from a survey methodology point of view, as only then can it be guaranteed that theoretical concepts are understood similarly across countries and their support or disagreement can be compared. Secondly, and this refers to actual social dynamics, it is important for a community to share similar understandings of fundamental values. For people to communicate across borders, it is invaluable to be able to refer to the same conceptual comprehensions. Thirdly, a common concept of transnational solidarity is important to ensure political legitimacy. If citizens and politicians come to understand the same regarding European solidarity, dialogue between citizens and politicians, both national representatives and those in Brussels, could improve.

Despite the events of the past decade transnational solidarity is relatively strong and the understanding of it is consistent across countries. The countries under consideration largely share the same ideas with regard to transnational solidarity. This notion seems reassuring, especially in view of the Covid-19 pandemic and its accompanying financial crisis. Although public support for transnational solidarity is supposed to vary from time to time, depending on political, economic, social and cultural conditions, the concept of transnational solidarity presented in this paper should still be relevant and help predicting public’s willingness to help others in the event that risk-sharing or redistribution is called for in future.

Further research could examine whether the concept of transnational solidarity presented here also applies when other indicators of EU-level risk-sharing and redistribution are integrated in the empirical model. In addition, a distinction between groups within countries could be a focus of research (see for instance Baute et al., 2018) or it could be tested whether or not this EU-related concept is also applicable to other international organizations and world regions. Even if the EU is unique in terms of cooperation between autonomous states, the two-dimensional concept of transnational solidarity could also exist elsewhere. This should be critically examined, especially with respect to comparability between countries. Alongside this, the two-dimensional concept identified may be used as a dependent variable in future studies. By applying structural equation models, respondents’ characteristics and underlying attitudinal correlations with regard to transnational solidarity could be uncovered.

The paper has argued for a shared concept of transnational solidarity across countries, even though the three EU nations investigated are known to be quite different with respect to public backing for European integration as well as the countries’ respective roles in previous European crises. However, since this study only analyzed three countries, the findings are not generalizable for all EU member states. This work could hence serve as a template for future studies analyzing transnational solidarity. In this respect, it would be particularly interesting to test the concept in EU accession countries of later enlargement rounds (e.g., Eastern European countries) or in countries that have survived previous European crises largely unscathed and are home to excellent welfare states (e.g., Denmark). In the light of previous research on solidarity within European societies, it seems reasonable to assume that the high level of comparability found in this study will become more difficult when the number of countries in any future sample increases (Lomazzi, 2021). In eastern European countries, for example, questions about an EU-wide welfare state could be interpreted differently as their communist pasts could have anchored a deviating idea of state protection in their minds. In northern European countries, in addition to diverging understandings of welfare state services, people’s interpretation of migration could also differ from those in other European areas. Due to their location far away from the external borders of southern Europe, the countries of Scandinavia are arguably less confronted with refugee migration and more concerned with intra-EU relocations. This is all pure speculation at this point and should be further investigated in future studies. As the present work has already examined a heterogeneous group of countries, though, the results are encouraging and a promising starting point for future EU-wide comparative studies.

## Appendix

See Figs. 2, 3, 4 and Tables 1, 2, 3, 4.

**Table 1:** Variable list

Dimension	Variable	Survey question	Recoded response categories
Risk-sharing	Financial assistance national bankruptcy	My country should provide financial assistance to other EU countries in the event of a national bankruptcy.	1 Totally disagree 2 Rather disagree 3 Rather agree 4 Totally agree
	Financial assistance natural disaster	My country should provide financial assistance to other EU countries in the event of a natural disaster (eg. floods, earthquakes).	1 Totally disagree 2 Rather disagree 3 Rather agree 4 Totally agree
	Financial assistance sharp increase incoming migrants	My country should provide financial assistance to other EU countries in the event of a sharp increase in incoming migrants in their territory.	1 Totally disagree 2 Rather disagree 3 Rather agree 4 Totally agree
Redistribution	EU promote solidarity	The EU’s aim should be to promote solidarity between member states.	1 Totally disagree 2 Rather disagree 3 Rather agree 4 Totally agree
	Reduce disparities between countries	The economic disparities between rich and poorer EU countries need to be reduced, even if this means that richer countries pay higher contributions.	1 Totally disagree 2 Rather disagree 3 Rather agree 4 Totally agree
	European welfare system	The EU needs a European welfare system for all European citizens, even if this means tax increases.	1 Totally disagree 2 Rather disagree 3 Rather agree 4 Totally agree

**Table 2:** Descriptive statistics (per country)

Variable	Abbreviation	Austria	Germany	Greece
Financial assistance national bankruptcy	sol_eco	N: 2742 mean: 1.898 sd: .885	N: 3341 mean: 2.274 sd: .817	N: 1086 mean: 2.951 sd: 1.089
Financial assistance natural disaster	sol_nat	N: 2799 mean: 3.040 sd: .791	N: 3564 mean: 3.416 sd: .651	N: 1182 mean: 3.698 sd: .629
Financial assistance sharp increase incoming migrants	sol_mig	N: 2734 mean: 2.027 sd: .946	N: 3348 mean: 2.340 sd: .878	N: 1146 mean: 2.412 sd: 1.181
EU promote solidarity	sol_aim	N: 2665 mean: 3.045 sd: .803	N: 3468 mean: 3.347 sd: .637	N: 1144 mean: 3.702 sd: .641
Reduce disparities between countries	red_eu	N: 2624 mean: 2.365 sd: .926	N: 3322 mean: 2.570 sd: .797	N: 1097 mean: 3.368 sd: .924
European welfare system	ess_eu	N: 2650 mean: 2.167 sd: .932	N: 3274 mean: 2.406 sd: .878	N: 1112 mean: 2.962 sd: 1.116
Risk-sharing	risk-s			
Redistribution	red			
*Additional sample characteristics*				
Age		15–29: N = 540(18.6%) 30–39: N = 464(16.0%) 40–49: N = 538(18.6%) 50–59: N = 591(20.4%) 60–69: N = 642(22.2%) 70–100: N = 122(4.2%)	15–29: N = 237(6.7%) 30–39: N = 367(10.4%) 40–49: N = 539(15.3%) 50–59: N = 881(25.0%) 60–69: N = 794(22.5%) 70–100: N = 707(20.1%)	15–29: N = 247(20.6%) 30–39: N = 212(17.7%) 40–49: N = 204(17.0%) 50–59: N = 181(15.1%) 60–69: N = 147(12.3%) 70–100: N = 207(17.3%)
Female		Female: N = 1448(50.2%) Male: N = 1439(49.9%)	Female: N = 1751(49.3%) Male: N = 1805(50.8%)	Female: N = 611(49.0%) Male: N = 587(51.0%)
Left–right self-placement (0: Left–10: Right)		N: 2565 mean: 4.880 sd: 2.170	N: 3493 mean: 4.718 sd: 1.911	N: 1104 mean: 5.602 sd: 3.131

Annotation: Instead of using a continuous variable representing respondents’ age, I opted for age categories as the original coding differed considerably between the countries. Moreover, since the educational level of respondents was surveyed differently in the respective countries, it is not possible to compare the groups with regard to this characteristic. Weighted presentation: for Germany and Austria, the weightings provided by the survey programs are applied, whereas the Greek sample is weighted by age and sex

**Table 3:** Correlation matrix

	Financial assistance national bankruptcy	Financial assistance natural disaster	Financial assistance sharp increase incoming migrants	EU promote solidarity	Reduce disparities between countries	European welfare system
Financial assistance national bankruptcy	1.0000					
Financial assistance natural disaster	AT: 0.32 DE: 0.31 GR: 0.07	1.0000				
Financial assistance sharp increase incoming migrants	AT: 0.62 DE: 0.55 GR: 0.21	AT: 0.39 DE: 0.33 GR: 0.15	1.0000			
EU promote solidarity	AT: 0.30 DE: 0.34 GR: 0.08	AT: 0.34 DE: 0.31 GR: 0.08	AT: 0.36 DE: 0.35 GR: 0.08	1.0000		
Reduce disparities between countries	AT: 0.45 DE: 0.46 GR: 0.06	AT: 0.29 DE: 0.27 GR: 0.07	AT: 0.47 DE: 0.44 GR: 0.09	AT: 0.47 DE: 0.44 GR: 0.25	1.0000	
European welfare system	AT: 0.47 DE: 0.41 GR: 0.11	AT: 0.28 DE: 0.23 GR: 0.06	AT: 0.48 DE: 0.39 GR: 0.10	AT: 0.45 DE: 0.37 GR: 0.30	AT: 0.61 DE: 0.61 GR: 0.28	1.0000

Annotation: For Germany and Austria, the weightings provided by the survey programs are applied, whereas the Greek sample is weighted by age and sex. AT: Austria (N = 2310); DE: Germany (N = 2806); GR: Greece (N = 887)

**Table 4:** Goodness of fit indices

Model	Chi-sq	df	CFI	∆ CFI	RMSEA	∆ RMSEA	SRMR	∆ SRMR
Configural (Austria)	94.424	8	0.983		0.061		0.026	
Configural (Germany)	133.888	8	0.977		0.066		0.032	
Configural (Greece)	3.495	8	1.000		0.000		0.010	
Baseline	231.808	24	0.981		0.058		0.028	
Metric	272.194	32	0.978	0.003	0.054	0.004	0.037	0.009
Scalar	814.807	40	0.928	0.050	0.087	0.033	0.069	0.032
Partial Scalar	402.122	36	0.966	0.012	0.063	0.009	0.048	0.011
*(Free intercepts sol_mig & sol_aim)*								

Annotation: Estimator: ML. Chi-sq. = chi-square (X^2^); df = degrees of freedom; CFI = comparative fit index; RMSEA = root mean square error of approximation; SRMR = standardized root mean square residual; **∆** fit index change relative to the previous model

## Footnotes

1 By this term, I refer to the period from 2010 onwards, when some EU member states were no longer able to service their public debt.

2 Lahusen and Grasso (2018) analyzed data collected by the TransSOL project in eight EU states in 2016/2017. They operationalize solidarity via questions on protest attendance, donation behavior as well as preferences for EU-wide fiscal and refugee solidarity. Their results show that solidarity is strongest concerning citizens within one’s own nation state, while solidarity concerning people from other EU countries is even lower compared to solidarity expressed towards people outside the EU.

3 Gerhards and colleagues (2019) rely on data collected by the Transnational European Solidarity Survey (TESS) in 13 EU member states in 2016 and distinguish between four domains of solidarity in the EU, namely fiscal solidarity, territorial solidarity, welfare solidarity, and refugee solidarity. According to their data, solidarity seems much stronger in the areas of fiscal, territorial, and welfare solidarity than one would have assumed from public reporting. Solidarity towards refugees, however, is much less widespread within the EU populace.

4 Inequality within the EU and between its member states and populations is still high and, based on recent research findings (for instance Schraff, 2020), is increasing. So redistribution policies continue to constitute an important pillar of transnational solidarity in the EU.

5 Despite the outstanding work of Baute et al., (2018; 2019a; 2019b) empirically examining the idea of Social Europe, the authors did not test for measurement invariance across countries. This may be due to a lack of publicly available cross-national survey data on the topic.

6 These different perceptions and interpretations can arise from many sources, ranging from the type of survey, the sample selection, and the translation of survey questions to different (and in part culturally conditioned) interpretations of terminology used (for instance see Lomazzi 2021).

7 A distinction is made between the three dimensions of local, global, and social solidarity. Comparing the overall concept of solidarity across borders is made particularly difficult by the dimension of global solidarity, which is therefore excluded in later steps of the analysis.

8 The EU aspect is only partially covered in Lomazzi’s dimension of global solidarity, as it involves a variable that records concerns about living conditions of fellow Europeans.

9 In Germany, the unemployment level in 2009 was 7.8% and continuously decreased over the years of crisis. In 2013 5.2% of the German population were registered as unemployed. In Austria, the unemployment rate in 2009 was 5.3%, reaching a low in 2011 with 4.6%. It then gradually increased to its pre-crisis level. For Greece there is a different picture. Whereas the state recorded an unemployment rate of 9.6% in 2009 it reached a level of 27.5% in 2013 (Eurostat, 2020b).

10 In Germany, the number of first-time asylum applicants rose from 172,945 in 2014 to 722,270 in 2016. The same applies to Austria. In 2014 the country had 25,675 asylum applicants and by 2015 the number had more than tripled. In contrast, for Greece the number of asylum applications rose every year from 2014 to 2019. Starting with a number of 7,585 in 2014, the country had 74,190 first-time asylum seekers in 2019 (a nearly tenfold increase) (Eurostat, 2020a). Consequently, in the Greek case migration pressures were ongoing when the survey was being undertaken.

11 For more information on the individual surveys and questionnaires, please see the published documentations of the respective polls.

12 I am aware that providing financial assistance is not the only possible sign of solidarity in a scenario of high migration, and previous studies have already studied other aspects of it (for instance Gerhards et al., 2019). However, granting financial assistance is, amongst other things, needed to help those countries accommodating refugees to care for new arrivals.

13 The German sample is on average older than the respondents in the other two countries. The Greek respondents are evenly distributed over the age groups and in Austria comparatively fewer older people were interviewed. These different age distributions could be due to the different survey modes used, as online surveys tend to reach younger people. With regard to sex, the samples are similar. When it comes to respondents expressed leanings on the left–right political spectrum, Greek respondents tend to be more right-wing compared to Austrian and German interviewees.

14 For more information on the method see for instance Olinsky et al. (2003) or Schafer and Graham (2002).

15 To test for configural measurement invariance, the variance of the latent variables is set to 1 to freely estimate the factor loadings of the indicators.

16 As an additional robustness check I conducted confirmatory factor analysis separately for each country and compared the results with an alternative model that consists of one rather than two latent factors. I compared two models with competing attitude structures by means of model-fit indices. Following this, for all the three countries my theorized model fits the data significantly better compared to the one-factor model.

17 In case the number of respondents is > 300, the following rules should apply for determining a good model fit: ∆CFI < 0.01, ∆RMSEA < 0.015, ∆SRMR < 0.03 (Chen 2007).

18 The chi-square difference test can provide significant results very quickly for large sample sizes. Therefore, in cases analyzing larger samples it is advisable to refrain from interpreting the chi-square values and instead to rely on other criteria governing the appropriateness of models (Chen, 2007).

## References

[CR1] Aichholzer, J., Partheymüller, J., Wagner, M., Kritzinger, S., Plescia, C., Eberl, J., Meyer, T., Berk, N., Büttner, N., Boomgaarden, H. & Müller, W. C. (2020). *AUTNES Online Panel Study 2017–2019 (SUF Edition)*. AUSSDA. DOI: 10.11587/QDETRI*(prerelease March 2020)*

[CR2] Baute S, Abts K, Meuleman B (2019). Public support for European solidarity: Between Euroscepticism and EU agenda preferences?. Journal of Common Market Studies.

[CR3] Baute S, Meuleman B, Abts K (2019). Welfare State attitudes and support for social Europe: spillover or obstacle?. Journal of Social Policy.

[CR4] Baute S, Meuleman B, Abts K, Swyngedouw M (2018). Measuring attitudes towards social Europe: A multidimensional approach. Social Indicators Research.

[CR5] Brown TA (2006). Confirmatory factor analysis for applied research.

[CR6] Byrne BM, Stewart SM (2006). Teacher’s corner: The MACS approach to testing for multigroup invariance of a second-order structure: A walk through the process. Structural Equation Modeling.

[CR7] Byrne BM, Shavelson RJ, Muthén B (1989). Testing for the equivalence of factor covariance and mean structures: The issue of partial measurement invariance. Psychological Bulletin.

[CR8] Chen FF (2007). Sensitivity of goodness of fit indexes to lack of measurement invariance. Structural Equation Modeling.

[CR9] Cieciuch J, Davidov E (2016). Establishing measurement invariance across online and offline samples. A tutorial with the software packages Amos and Mplus. Studia Psychologica.

[CR10] Cieciuch J, Davidov E, Schmidt P, Algesheimer R, Wolf C, Joye D, Smith TW, Fu Y (2016). Assessment of cross-cultural comparability. The SAGE handbook of survey methodology.

[CR11] Davidov E, Datler G, Schmidt P, Schwartz SH, Davidov E, Schmidt P, Billiet J (2011). Testing the invariance of values in the Benelux countries with the European Social Survey: Accounting for ordinality. European Association for Methodology series. Cross-cultural analysis: Methods and applications.

[CR12] Davidov E, Meuleman B, Cieciuch J, Schmid P, Billiet J (2014). Measurement equivalence in cross-national research. Annual Review of Sociology.

[CR13] Davidov E, Cieciuch J, Meuleman B, Schmidt P, Algesheimer R, Hausherr M (2015). The comparability of measurements of attitudes toward immigration in the European Social Survey: Exact versus approximate measurement equivalence. Public Opinion Quarterly.

[CR14] Della Porta D (2018). Solidarity mobilizations in the ‘refugee crisis’. Contentious Moves.

[CR15] Díez Medrano J, Ciornei I, Apaydin F, Recci E, Favell A, Apaydin F, Barbulescu R, Braun M, Ciornei I, Cunningham N, Díez Medrano J, Duru DN, Hanquinet L, Pötzschke S, Reimer D, Salamońska J, Savage M, Solgaard Jensen J, Varela A (2019). Explaining supranational solidarity. Everyday Europe. Social transnationalism in an unsettled continent.

[CR16] Domurath I (2013). The three dimensions of solidarity in the EU legal order: Limits of the judicial and legal approach. Journal of European Integration.

[CR17] Esarey J, Salmon T, Barrilleaux C (2012). Social insurance and income redistribution in a laboratory experiment. Political Research Quarterly.

[CR18] ESM (2020). ESM Treaty - consolidated version (all official languages of ESM Members). Available at: https://www.esm.europa.eu/legal-documents/esm-treaty (accessed 01 July 2020).

[CR19] European Commission (2020). European Structural and Investment Funds. Available at: https://ec.europa.eu/regional_policy/en/funding/ (accessed 01 July 2020).

[CR20] Eurostat (2020a). Asylum and first time asylum applicants. Annual aggregated data (rounded). Available at: https://ec.europa.eu/eurostat/databrowser/view/tps00191/default/table?lang=en (accessed 01 July 2020a).

[CR21] Eurostat (2020b). Total unemployment rate. Available at: https://ec.europa.eu/eurostat/databrowser/view/tps00203/default/table?lang=en (accessed 01 July 2020b).

[CR22] Ferrera M (2017). The Stein Rokkan Lecture 2016. Mission impossible? Reconciling economic and social Europe after the euro crisis and Brexit. European Journal of Political Research.

[CR23] Ferrera, M. & Pellegata, A. (2017) Can Economic and Social Europe Be Reconciled? Citizen Views on Integration and Solidarity*.* Università degli Studi di Milano, *REScEU Working Paper*.

[CR24] Genschel, P. & Hemerijck, A. (2018). Solidarity in Europe. European University Institute, *School of Transnational Governance Policy Briefs* 2018/1.

[CR25] Gerhards J, Lengfeld H, Häuberer J (2016). Do European citizens support the idea of a European welfare state? Evidence from a comparative survey conducted in three EU member states. International Sociology.

[CR26] Gerhards, J., Lengfeld, H., Ignácz, Z., Kley, F. K. & Priem, M. (2018). How strong is European solidarity? *BSSE Working Paper* 37.

[CR27] Gerhards J, Lengfeld H, Ignácz Z, Kley FK, Priem M (2019). European solidarity in times of crisis. Insights from a thirteen-country survey.

[CR28] GESIS (2020) GESIS Panel Standard Edition. GESIS Datenarchiv, Cologne. ZA5665 Datenfile Version 35.0.0. 10.4232/1.13425*(prerelease November 2019)*

[CR29] Grimmel A (2021). “Le Grand absent Européen”: Solidarity in the politics of European integration. Acta Politica.

[CR30] Grimmel A, Giang SM, Grimmel A, Giang SM (2017). Introduction: Solidarity lost? The European Union and the crisis of one of its core values. Solidarity in the European Union: A Fundamental Value in Crisis.

[CR31] Hooper D, Coughlan J, Mullen MR (2008). Structural equation modelling: Guidelines for determining model fit. Electronic Journal of Business Research Methods.

[CR32] Ignácz Z (2021). Similarities between European and National Solidarity. An empirical thought experiment applied to 13 European Countries on attitudes towards redistribution. Sozialpolitik Ch..

[CR33] Kankaraš M, Moors G (2009). Measurement equivalence in solidarity attitudes in Europe insights from a multiple-group latent-class factor approach. International Sociology.

[CR34] Katsanidou A, Reinl AK (2020). Solidarity and Populism: A data collection project. SowiDataNet Datorium Data File Version.

[CR35] Katsanidou A, Reinl AK, Eder C (2021). Together we stand? Transnational solidarity in the EU in times of crises. European Union Politics.

[CR36] Kleinke K, Schlüter E, Christ O (2017). Strukturgleichungsmodelle mit Mplus: Eine praktische Einführung.

[CR200] Lahusen C (2016) Transnational solidarity within the European union: Towards a Framework of Analysis [Paper presentation]. In: *8th Pan-European Conference on the European Union*, Trento, 15-18 June 2016.

[CR37] Lahusen, C. (2020). European solidarity: an introduction to a multifaceted phenomenon. In *Citizens’ Solidarity in Europe*. Cheltenham, UK: Edward Elgar Publishing, pp. 1–28.

[CR38] Lahusen C, Grasso M, Lahusen C, Grasso M (2018). Solidarity in Europe: A comparative assessment and discussion. Solidarity in Europe Citizens’ responses in times of crisis.

[CR39] Lomazzi V (2021). Can we compare solidarity across Europe? what, why, when, and how to assess exact and approximate equivalence of first- and second-order factor models. Frontiers in Political Science.

[CR40] Olinsky A, Chen S, Harlow L (2003). The comparative efficacy of imputation methods for missing data in structural equation modeling. European Journal of Operational Research.

[CR41] Pantazatou K (2015). Promoting solidarity in crisis times: Building on the EU Budget and the EU Funds. Perspectives on Federalism.

[CR42] Pettersen PA, Borre O, Scarbrough E (1998). The welfare state: The security dimension. The scope of government.

[CR43] Reinl AK, Baldassari M, Castelli E, Truffeli M, Vezzani G (2020). Euroscepticism in times of European crises: The role of solidarity. Anti-Europeanism, critical perspectives towards the European Union.

[CR44] Roller E, Borre O, Scarbrough E (1998). The welfare state: The equality dimension. The scope of government.

[CR45] Rothstein B (1998). Just institutions matter: The moral and political logic of the universal welfare state.

[CR46] Sangiovanni A (2013). Solidarity in the European Union. Oxford Journal of Legal Studies.

[CR47] Sangiovanni, A. (2018). Solidarity [Unpublished manuscript]. In: *Workshop “Understanding Solidarity—New Challenges, New Approaches Philosophy”*, Hamburg, 25–27 January 2019.

[CR48] Schafer JL, Graham JE (2002). Missing data: Our view of the state of the art. Psychological Methods.

[CR49] Schelkle W (2017). The political economy of monetary solidarity: Understanding the Euro experiment.

[CR50] Schraff D (2020). Is the member states' curse the EU's blessing? inequality and EU regime evaluation. JCMS: Journal of Common Market Studies.

[CR51] Schulmeister, P., Büttner, M., Chiesa, A., Defourny, E., Hallaouy, S., Maggio, L., Tsoulou-Malakoudi, D., Friedli, M. E. & van Gasse, B. (2019). *Closer to the citizens, closer to the ballot. Eurobarometer Survey 91.1 of the European Parliament A Public Opinion Monitoring Study*. European Parliament Directorate-General for Communication Public Opinion Monitoring Unit.

[CR52] Steenkamp JBE, Baumgartner H (1998). Assessing measurement invariance in cross-national consumer research. Journal of Consumer Research.

[CR53] Steinvorth U, Grimmel A, Giang SM (2017). Applying the idea of solidarity to Europe. Solidarity in the European Union: A fundamental value in crisis.

[CR54] Stjernø S (2011). The idea of solidarity in Europe. European Journal of Social Law.

[CR55] Vandenbroucke F (2017). Risk reduction, risk sharing and moral hazard: A vaccination metaphor. Intereconomics.

[CR56] Vandenbroucke, F. (2018). Social policy in a monetary union: puzzles, paradoxes and perspectives. In: Boone M, Deneckere G and Tollebeek J (eds) *The End of Postwar and the Future of Europe—Essays on the work of Ian Buruma*. Verhandelingen van de KVAB voor Wetenschappen en Kunsten. Nieuwe reeks, 33, Uitgeverij Peet.

[CR57] Vandenbroucke, F. (2020). Solidarity through Redistribution and Insurance of Incomes: The EU As Support, Guide, Guarantor or Provider? *The Amsterdam Centre for European Studies Research Paper* 2020/01*.*

[CR58] Wallaschek S (2020). The discursive construction of solidarity: Analysing public claims in Europe’s migration crisis. Political Studies.

